# Attributions of Loneliness—Life Story Interviews with Older Mental Health Service Users

**DOI:** 10.3390/healthcare12111133

**Published:** 2024-05-31

**Authors:** Annette Burns, Gerard Leavey, Brian Lawlor, Jeannette Golden, Dermot Reilly, Roger O’Sullivan

**Affiliations:** 1 Institute of Public Health, D08 NH90 Dublin, Ireland; 2 Bamford Centre for Mental Health & Wellbeing, Ulster University, Coleraine BT52 1SA, UK; 3 Global Brain Health Institute, Trinity College, D02 PN40 Dublin, Ireland; 4 Martha Whiteway Day Hospital, Mercer’s Institute for Successful Ageing, St. James’s Hospital, D08 KC95 Dublin, Ireland

**Keywords:** loneliness, life course, qualitative

## Abstract

There is growing evidence on the prevalence and impact of loneliness, particularly among older people. However, much less is known about the personal origins of loneliness and how it persists, or not, over an individual’s life course. This study aimed to increase understanding of the personal experiences of loneliness among older adults across the life course. Central to this study was giving voice to the participants and allowing them to define loneliness, what it meant to them, and how it affected them throughout their lives. This qualitative study employed 18 life story interviews with older adults attending a mental health service. We explored their personal experiences of loneliness and the situations and factors associated with loneliness across the life course. We identified three distinct typologies of loneliness: those who experienced (1) chronic loneliness since childhood, (2) chronic loneliness after a life-changing event in midlife, and (3) loneliness which remained situational/transitional, never becoming chronic. This study found the seeds of chronic life course loneliness are often determined in childhood. Early detection and intervention may prevent situational loneliness from becoming chronic. More research is needed from a life course approach to help understand and address the causes and consequences of loneliness.

## 1. Introduction

Loneliness is a personal, subjective negative experience or perception of lacking qualitatively or quantitatively meaningful connections with an intimate other; family and/or friends; or community life [1]. It is a complex multidimensional construct with different types of loneliness—social loneliness describes the lack of social connections; emotional loneliness describes an absence of a close emotional connection; and existential loneliness describes the feeling of being ‘cut off’ or alienated from others and society in general. There are also different frequencies of loneliness—transitional loneliness is associated with a time of change, for example, bereavement or retirement, whereas situational loneliness is associated with certain events or contexts such as the public health social measures during the COVID pandemic; transient or occasional loneliness describes a feeling that comes and goes; and finally, chronic or persistent loneliness is where one feels lonely all or most of the time. Loneliness is associated with a range of poor health outcomes, including depression and anxiety, non-communicable diseases such as cardiovascular disease, negative health behaviors (including smoking), frailty, and, ultimately, premature mortality [2]. Chronic or persistent loneliness has been found to have the greatest negative health outcomes [3]. Despite loneliness being a significant risk factor for poor health outcomes, too often our understanding, conceptualization, and research studies are narrow in their focus. This is in part due to the traditional perception of loneliness as being a social problem of old age, which in turn framed much of the work in this area and has hindered the evolution of this area not only theoretically but methodologically.

Hawkley and Cacioppo [4] suggest that up to 80% of those under 18 years of age and 40% of those aged 65+ report being lonely at least some of the time. In spite of documented high rates of loneliness reported by adolescents and young adults [5,6], research to date has not sufficiently addressed loneliness across all stages of the life course [7] but instead tended to focus on segments, for example, either young people or older people. This is significant within the context of this paper in that a life course approach in qualitative research allows the individual to reflect across their life experiences of what they deemed to be the causes and consequences of their loneliness. This offers a contrast to many of the existing qualitative studies conducted with older adults which tend to focus on exploring their current loneliness [8,9,10,11,12,13,14,15]. A recent qualitative systematic review, which included papers on those aged from 7 to 103, found three common features to loneliness—it was psychologically mediated but contextually grounded, it was centered on painful disconnection, and finally it was associated with a lack or loss. However, as noted within the limitations of that review, it did not include any studies exploring loneliness in specific clinical or adjacent populations [7].

The prevalence of loneliness among people with mental health difficulties has been well established with rates as high as 80% observed among those with severe mental illness such as psychosis [16] and strong associations demonstrated for anxiety, social anxiety, suicidal ideation, and in particular depression [17,18,19,20]. Loneliness has also been qualitatively explored among these populations, with studies investigating loneliness among young and middle-aged adults with mental health difficulties [21,22,23].

Overall, there remains a dearth, however, of studies exploring the qualitative experience of loneliness among older adult psychiatric populations in relation to both their current experiences of loneliness, as well as across their life course [24]. One recent study explored loneliness in older adults with mental health problems and found service users emphasized their thoughts and feelings rather than behaviors when discussing loneliness. This led the authors to conclude that greater awareness of psychological dimensions of loneliness in older adults with complex mental health problems is important [25]. The current study offers the opportunity to further explore loneliness in an older adult population with mental health difficulties in terms of what is important from their perspective, as well as to capture this from a life course perspective rather than only in their current older age. The aim of this study was therefore two-fold, first to address knowledge gaps relating to qualitative studies of loneliness in older adults using mental health services and secondly to increase our understanding of the personal experiences of loneliness across the life course among this population and particularly the factors that may be associated with their experiences of loneliness.

## 2. Materials and Methods

### 2.1. Study Setting and Data Collection

This study was conducted in 2021 at a day hospital and associated community service providing a mental health service for older adult patients with disorders, including depression, anxiety, and psychosis, within a catchment area of approximately 20,000 older people in Dublin, Ireland. Services were provided by a multi-disciplinary team including psychiatrists, nurses, occupational therapists, social workers, and psychologists and included individual and group therapy, as well as skills training.

#### 2.1.1. Sample Selection

Local gatekeepers (community mental health nurses or ward nurses or designated reference health professionals) were briefed by the project researcher (A.B.) before approaching patients in January 2021 with brief self-completion questionnaires which collected data, including basic socio-demographic information (age in years, gender, education, occupation, whether living alone and marital status), loneliness (as assessed by the 11-item De Jong Gierveld Loneliness scale [26] and a single item in the CES-D-R 10 depression scale) [27,28], and willingness to participate in a qualitative interview. These two measures were selected to identify objective loneliness within the current study as they are commonly used scales and have established reliability.

The De Jong Gierveld Loneliness scale is widely used to assess loneliness and was developed for use with older adults [26,29]. Its reliability has been demonstrated with a mean internal consistency reliability coefficient of 0.84, 95% CI [0.83, 0.86] based on meta-analysis of 94 effect sizes, with higher reliability seen in clinical samples such as those with mental health problems [29]. The creators of the scale recommend a cut-off point of 3 to distinguish between lonely and not lonely people [30].

The CES-D-R 10, which has been shown to have strong psychometric properties in a psychiatric sample [27], was included as depressive symptoms were of interest as part of the larger study, as well as to provide an additional single-item assessing loneliness. Single-item measures such as that included in the CES-D also perform well in assessing loneliness and are the most commonly used measures of loneliness [31], which increases comparability. Moreover, while the De Jong Gierveld scales asks about general feelings of loneliness, the CES-D-R 10 asks about the past week. In the current study, both chronic and current loneliness were of interest, and we wanted to ensure patients reporting either were eligible for interview.

Eligible participants for the pre-interview self-completion questionnaire were older adults currently attending the day hospital or under the care of the community mental health team, who were English-speaking and provided written informed consent. Gatekeepers were asked to recruit participants face to face or via telephone by providing the self-completion questionnaire in-person or via post to all willing patients and to not purposefully select those they suspected would meet loneliness thresholds. Given the prevalence of COVID-19 at that time and associated public health social measures, some patients were being reviewed remotely and were therefore recruited remotely by phone with staff posting self-completion questionnaires out to their homes. From January–March 2021, 30 questionnaires were returned with 25 of these participants reporting they were willing to participate in a qualitative life story interview exploring loneliness across the life course. All 25 also met minimum loneliness criteria scoring 3 or more on the De Jong Gierveld Loneliness Scale [26] or reporting they ‘felt lonely’ at least ‘3–4 days’ a week in response to CES-D-R 10 item 9 [27] (in line with our purposive sampling approach) and were contacted between March and June 2021. Notably, although all 25 were objectively assessed as being lonely, some participants indicated they did not presently feel lonely at the time of the interviews. There was also at times discord between questionnaires and interviews in relation to the life stage at which participants first recalled feeling lonely. Given our focus on giving voice to the participants and allowing them to define loneliness, what it meant to them, and how it affected them throughout their lives, findings are presented in this paper in line with how the participants narrated their own stories in relation to life course loneliness during their interview.

Following multiple attempts to contact all 25 participants, we were able to interview 18 people, with the remaining 7 participants no longer contactable to schedule or complete an interview with no other reasons for non-participation provided. There is no consensus on the required number of interviews in qualitative research, which can vary according to target group, subject matter, and method of interviewing [32]. In this study, data saturation was achieved with the 18 participants and no further recruitment was required.

#### 2.1.2. Life Story Interviews

Due to COVID-19 public health restrictions, we used telephone interviews rather than face-to-face interviews, as originally planned. All participants were interviewed once and without other non-participants present on the call. Brief field notes were made during the call to guide the interview only rather than informing later analysis. The interviewer, A.B., was a female PhD graduate employed at the time as Interventions Officer at the Institute of Public Health. A.B. had a background in psychology and was experienced in conducting interviews with people of all ages and circumstances, including those currently in inpatient psychiatric care as well as on sensitive and potentially painful topics. No relationship with the interviewer was established prior to study commencement. Participants only received a phone call from the interviewer to schedule or complete the interview. Participants knew the interviewer was a researcher and knew the research was being conducted to better understand experiences of loneliness across the life course. The information leaflet also informed in relation to the interviewer’s experience, as mentioned above, in conducting qualitative interviews with people of all circumstances and on sensitive topics.

Interviews (ranging from 19 to 114 min and an overall average of 45 min) were conducted using a life story approach which explored interviewees current situation socially before asking them about loneliness across all life stages, starting with their first recalled experience and then finished up by exploring some ways of responding to or coping with loneliness (see Supplementary Materials File S1 for interview guide). The interview guide was piloted in advance and was also reviewed by A.B. and R.O. after the first three interviews. No changes were made to the guide following piloting or initial interviews. This was not surprising to the researchers given the guide takes a very open approach, including only brief prompts. Significant time was also spent developing the guide prior to piloting, with A.B. attending an additional Narratives and Storytelling in Qualitative Research course in preparation for the study.

Narrative qualitative designs involving in-depth life story interviews have been described as offering rich insights into the lived experience, the meaning of personal experience, and the trajectory of life across time [33]. Narrative inquiries have also been specifically recommended in exploring the lived experience of loneliness, especially since self-labelling of the personal experience of loneliness can be rare to non-existent [34]. The adoption of a narrative, qualitative approach in the current study therefore allowed an in-depth exploration of individuals’ relationships and experience with loneliness throughout their lives, including trigger points, duration of loneliness and coping mechanisms. A debrief was conducted with each participant post interview also to ensure they were not distressed by the experience. Interviews were digitally recorded and transcribed verbatim with identifying data removed and pseudonyms assigned for anonymity. Participants were reminded, as per the information leaflet, that they could review interview transcripts should they wish, but no participant ultimately opted to do this. They were also advised that they could review a summary of all findings at the end of the research, but none requested this.

The methodological orientation underpinning this qualitative study was Phenomenology given our aim to understand the unique lived experience of life course loneliness for individuals [35]. It focused on giving voice to the participants to share their lived experiences of loneliness and own understanding of their experiences, feelings, associated meaning, and perceptions of loneliness across their life. We employed the consolidated criteria for reporting qualitative research (COREQ) [36] to ensure thorough reporting of our study and this checklist is available as a Supplementary Materials File S2.

### 2.2. Analysis

Data were transcribed and then analyzed using inductive thematic analysis [37] with all coding completed using NVivo 12. Each interview was coded for loneliness by life stage and by associations or key factors in the loneliness experienced as described by the interviewee, as well as the other things that were happening in their life at those times. A.B. conducted the coding of all transcripts and mapping of themes and sub-themes. A second researcher (R.O.) listened to all the interview recordings and independently reviewed a random sample (*n* = 9) of coded transcripts to ensure appropriateness of the codes, themes, and sub themes. Any differences were discussed, and a consensus was reached. Once all data had been coded, the research team were able to identify 3 key typologies or groups in relation to life course loneliness:

- Group 1, who were chronically lonely from childhood (*n* = 7).
- Group 2, who became chronically lonely in midlife; i.e., they did not really recover from some specific change in midlife and still feel lonely now (*n* = 4).
- Group 3, for whom loneliness was situational or transitional only and never became chronic (*n* = 7).

For each group, overarching themes were identified in relation to their experience of loneliness across their life course. The typology and these themes were reviewed and refined to ensure each theme was based on coded data which formed a coherent pattern and that key themes identified were valid in their reflection of each subgroup as a whole [31].

## 3. Results

Participants were adults 18 or older, ranging from 67 to 84 years (with one participant not providing age (mean age 76)). The sample included 12 women, 1 transwoman, and 5 men. A third of the participants were cohabiting or married (*n* = 6) and ten were living alone at time of interview. Four participants had been educated to degree/diploma level, seven had completed secondary or high school, while the remaining had completed primary level education only. Following inductive thematic analysis, three distinct groups or typologies of loneliness were identified in relation to life course loneliness in the current study. The first group were chronically lonely throughout life, with issues from childhood onwards linked to loneliness and reports of loneliness at every stage of life from childhood onwards. For the second group, who tended to rely on a small network, their situational/transitional loneliness became chronic after they failed to fully recover from some specific change in midlife and still feel lonely now. For the third group, loneliness remained situational/transitional only and never became chronic.

The themes around life course loneliness identified for each of these three groups are present below, while Table 1 also provides an overview of the three loneliness typologies identified, alongside themes identified in each group and illustrative quotation.

### 3.1. Group 1: Chronically Lonely since Childhood

Interrupted and dysfunctional parental bonds, difficulties making and maintaining close friendships, alcohol as a coping mechanism, inability to be authentic self or connect with others, and later life acceptance were all themes in group one (*n* = 7), those chronically lonely since childhood.

#### 3.1.1. Interrupted and Dysfunctional Parental Bond/Attachment

##### Bereavement and Separation

Separation from or loss of a parent in childhood was prevalent among this group with both John and Thomas losing their fathers and Martin losing both parents, as well as his guardian aunt. Beyond bereavement, separation was also described with a years-long hospitalization for Alex as well as a father forced to work away from home and John also seeing his father only rarely before his passing:

*“He died when I was still quite young, I was just turned 12 when he died and I can’t say I saw him three or four times in my young life”.*

[John]

##### A Dysfunctional Relationship with Parents

There were also reports of poor relationships with parents/guardians, where they were present with reports of violence and alcohol abuse in the home, as well as fear around dominant maternal figures:

*“Oh no, I wouldn’t talk to her [grandmother]. Because you see, I’d be afraid to talk to her…But it was like, those two, the granny and my mother, they were ruling the roost”.*

[Patricia]

For John, this dominance from his mother was in the form of stopping him from socializing with other children, which he felt also left him lacking in social skills as a teenager (Table 1). Ellen, meanwhile, described being restricted socially to the family home throughout life as a mutual choice:

*“I was always at home with my mam and dad and my sister …because they were more important…and I was happy with what I was doing”.*

[Ellen describing her social network in midlife]

Ellen noted, however, being overly worried as a child, that something would happen to her parents and also identified the role of this over attachment in her ongoing ‘co-dependence’ and lack of friends outside her family.

#### 3.1.2. Close Friendships Difficult or Lacking

Difficulties in making and maintaining close friendships, as children and throughout life, were also common among those chronically lonely. For some, family seemed to represent the only friendships they had and Alex described difficulties relating to the other boys as a child and this persisted throughout life.

*“I hated going back to the boys …I had one friend. I had a friend who was a lovely girl. I used to know her until she died … I just enjoyed the girls’ company rather than anyone else [as a young adult]”.*

[Alex]

#### 3.1.3. Alcohol as a Coping Mechanism

For Martin, lifelong shyness also made friendships a challenge. It was only through the use of alcohol to help overcome his shyness and a longer period of time that he was finally able to establish closer friendships with workmates, having previously struggled at school in the context of bullying and shyness:

*“I started to have a jar [alcohol] there and of course that’s part and parcel of the scene. Maybe that was one way of doing it, to not be as shy… in latter years it became a problem alright”.*

[Martin]

Alex also described overcoming her shyness and loneliness with alcohol and had leant on alcohol again recently (Table 1). John and Thomas described withdrawing into alcohol, with John using alcohol to pass the time and to cope with his ‘situation’ in terms of depression and loneliness, while Thomas started drinking to be ‘one of the lads’ and avoid the ‘boredom and loneliness’ of being at home, which affected his sport and later his family life as he felt an inability to stay at home and connect with them.

#### 3.1.4. Inability to Be Authentic Self or Connect with Others

An inability to be their authentic self or connect with others was observed across this group. For some, this was simply expressed as an inability to connect, while for others it was a difficulty in speaking up at all,

*“You’d be kind of afraid to say anything … I do often wonder with people or I admire people and the way they’re able to talk and they don’t see they’ve any hindrance in speaking up … That was one of the things I didn’t like about myself”.*

[Martin]

or in confiding in or trusting people and achieving genuine closeness:

*“I’m a great chatterbox—in a group, I would contribute lengthy. But I never had that real closeness”.*

[Patricia]

Alex and Colette both spent many years unable to be their authentic selves due to coming to terms with her own trans identity for Alex, which was associated with ‘huge amounts of loneliness’, and an abusive, controlling marriage for Colette. Colette describes herself as better now with the end of this marriage and feels she is now ‘a good mixer’, while Alex describes greater self-acceptance and comfort and how mental health staff have helped her to be herself.

#### 3.1.5. Later Life Acceptance

Alex and Colette were not alone in reporting a later life sense of achieving greater acceptance or peace. At the time of interview, Patricia (although meeting objective thresholds) reported she no longer felt loneliness and described a sense of acceptance of herself and her life at this stage,

*“It just all seemed to fall into place after a while, it took me a while. But I don’t feel lonely now…And then you’re also more at peace with yourself, that’s the other thing. I find you’re more accepting of yourself… I realize, there is no perfect life”.*

[Patricia]

Martin also described being better than he was before,

*“I try to do the best I can every day. And I’m lucky that I have a family around me that looks after me... you say to yourself, God, aren’t you lucky? You know what I mean?”*

[Martin]

and John, Thomas, and Martin had all recovered from their use of alcohol and were now sober.

### 3.2. Group 2: Became Chronically Lonely after a Life-Changing Event in Midlife

For all those in the second group (*n* = 4), a significant event in midlife from which they did not seem able to fully recover led them to chronic loneliness. For all of these individuals’, midlife was also their first experience of loneliness, with no loneliness as a child, teenager or young adult.

#### 3.2.1. A Life Changing Event

In the case of Marianne and Elizabeth, this change was first related to their marriages, with Marianne’s husband leaving her and Elizabeth’s husband passing away, although Elizabeth also experienced two other losses in midlife which she struggled to process:

*“When my husband died...I think I just really didn’t grieve enough…. I just didn’t have time for it…When my father died, I was working so I didn’t even get time for that... my friend died as well as I was kind of working”.*

[Elizabeth on several losses in midlife and not making time to grieve]

For David, the experience of a mental health episode and the isolation around that led to his first experience of loneliness (Table 1), while for Paula, it was the loss of both of her parents and a close lifelong friend in quick succession when she was in her 40s:

*“I suppose the first time I experienced loneliness was when my mother died, and then my father died five months later… that was a double whammy… I had one particular friend since I was a toddler, but she died in her early forties so again”.*

[Paula on multiple losses in her 40s]

#### 3.2.2. Clusters of Losses/Complicated Grief

Clusters of loss were a feature for all three women in group two, with Marianne also reporting a number of losses over a longer period, when several years after her husband left, her mum passed away, a family tragedy occurred, she gave up both her dogs, and she also lost two sisters, as well as friends.

A theme of not being able to truly recover or get over these clusters of loss and bereavements was identified, with Elizabeth, as above, describing not taking time to process her grief, while Paula also struggled to get over her losses and spoke about specific losses she could not recover from or replace, including her mother and a close friend:

*“My last friend died four years ago. And I never really recovered from that, she was a really, really close friend… I never quite got over it, I think of her every day [Paula on her mother] …And I suppose that I have no one new to replace those close people that I was close to. They’re all dead”.*

[Paula]

Marianne also described feeling each loss she suffered very deeply and describing herself as ‘a very soft person’. She missed her mother terribly, ‘cried and cried’ over her dogs, was ‘broken-hearted’ over her sister, and also took it ‘very bad’ when her husband left.

While only reporting one loss, on the other hand, David did acknowledge the loneliness around his wife being gone, having passed away three years ago and his really feeling this gap when out with others and their partners.

#### 3.2.3. Reliance on a Small Network

There was also a tendency among this group to be more introverted, to place great value on key relationships and to prefer a smaller network. While no difficulties connecting or making friends were reported, it was a choice to often focus on just a few people. Both David (Table 1) and Paula described themselves as preferring to focus on one or two friends and avoid crowds and parties:

*“I was always with lots of friends. But the girl I mentioned to you there that died in her forties, she was my best friend in school…and we were always together during the day. So, I’ve always been a sort of one person at a time, I’m not very comfortable in groups”.*

[Paula]

A choice Paula now advises against and feels is dangerous:

*“It’s not a good idea, I wouldn’t recommend it to any young person today, I think you should have a group or acquaintances or whatever. I think it’s a bad thing to have all your eggs in one basket, it’s dangerous”.*

[Paula]

Both Marianne and Paula also expressed being incredibly close to their mothers and closer it seems than other siblings were, with these losses then particularly hard for them to experience.

*“Me and [my mother] used to look at the telly at night because my husband used to work at nighttime you know and we used to go shopping together and we’d pick out things for the house”*

[Marianne]

### 3.3. Group 3: Loneliness Remained Situational/Transitional Only and Never Became Chronic

For the third group (*n* = 7), loneliness remained situational/transitional only throughout the life course and never became chronic. The themes in this group included periods of transitional loneliness around bereavements and losses, loneliness around being on your own, and fleeting loneliness related to clear contextual factors. For this group, loneliness appeared in relation to clear environmental prompts and was relatively short-term each time.

#### 3.3.1. Periods of Transitional Loneliness around Bereavements and Losses

The most common source of loneliness among this group was bereavement and loss, with the loneliness felt in response in most cases seeming fairly temporary and not prolonged or complicated given the context:

*“I was just about 16 when my dad died ...I suppose it was loneliness”.*

[Dolores describing some mild loneliness around her father’s death]

Peter similarly described loneliness when he lost his father, with his next recalled loneliness after this being around the loss of his mother:

*“The next time I remember was about 10 or 12 years afterwards, my mother died. That was kind of the same thing”.*

[Peter]

These interviewees all, however, were able to cope:

*“I’d get out in the garden, I’d do something… I used to love reading and I’d read a book”*

[Angela]

and reported a recovery from or resolution to this loneliness rather than the loss transitioning them into chronic loneliness.

#### 3.3.2. Being on Your Own—Siblings, Separation, and Being Left behind

There was also a trend among this group in relation to isolation from siblings or lack of siblings being associated with some loneliness at various life stages.

Rebecca recounted childhood time with no siblings as a possible time of loneliness for her:

*“…my other sister wasn’t born until I was seven. So, there was that period of being on my own. Now I wasn’t conscious of loneliness or anything like that but…”*

[Rebecca]

while Tina at times missed her siblings when she went to live in the countryside to attend another school as they were separated, but again, this feeling would pass and they still remained close (Table 1).

Jane also noted how being the youngest left her somewhat isolated from the others and temporarily experiencing some loneliness as a teenager when her siblings had all left home and married (Table 1), although she ‘always had friends at school’ and generally was rarely lonely.

For Angela and Peter, this period before they themselves got married also seems to have been a time of some transitional loneliness, though in their case there was more a sense of being left behind by friends at this stage rather than siblings. Angela described getting a dog due to loneliness before she herself got married, while Peter recalled how young adulthood was a time where ‘core friends’ would be lost to migration and marriage (Table 1).

#### 3.3.3. Fleeting/Contextual Loneliness

Among group three, an overarching tendency towards experiencing situational loneliness, which was largely fleeting, in response to contextual factors or circumstantial barriers was identified. These factors were diverse and included the pandemic; the health and mobility of the interviewee and those close to them; finances; being away from family; caring responsibilities; and experience of prejudice/bullying.

All four who reported in the interview that they were currently experiencing loneliness mentioned the impact of the pandemic:

*“Before this COVID … there was loads of little clubs going around for the older person, and bowling, outdoor bowling, indoor bowling, all of those things were there in the clubs and you could join them if you wanted to. It cost a small bit of money but not very much and you’d meet people and that sort of thing”.*

[Dolores]

The COVID-19 pandemic, for Rebecca, represented the only clear time of loneliness in her life she could definitively recall. She indicated she was content to live alone before the pandemic, when she was busy and had places to go (Table 1).

The health of others impacted at times, such as for Peter, when his wife was in hospital and for Nora, whose dance partner became ill with cancer:

*“…I did a lot of set dancing up to the age of 60/65 and then my friend got cancer. So, that kind of put a stop to that”*

[Nora]

For Jane, temporary loneliness was experienced with her son and only child starting school, while Tina felt lonely when living in Spain for a period as a young adult.

Both finances and prejudice caused Dolores loneliness at times, with her inability to join her friend on holidays as a teenager,

*“The difference between my friend and myself was she had other friends that she worked with and she would go off with them on holiday, which I couldn’t afford to go. Now that would be about the most loneliness I would have suffered”*

[Dolores]

and the experience of prejudicial bullying as a child and teenager (Table 1), although Dolores was able to move on from this and did not experience loneliness related to prejudice as she got older.

As above, these contextual factors which were external were mostly responded to with fleeting loneliness which never became chronic, although in one or two cases where the contextual factor itself was not resolving over time, it continued to be associated with some loneliness, such as in the case of continuing to care for her husband for Jane and the continuing deterioration of her eyesight for Dolores, who loves baking and embroidery:

*“They did say I could possibly go blind completely, but I’m praying that I won’t, but if it happens, I’ll just have to take it, and that’s it… It’s the loneliness comes in with my lack of eyesight”.*

[Dolores]

## 4. Discussion

The aim of this study, using life history interviews with older patients currently attending mental health services, was to increase the understanding of the personal experiences of loneliness across the life course and particularly the factors that may be associated with their experiences of loneliness.

Following thematic analysis, three distinct groups or typologies of loneliness were identified in relation to life course loneliness in the current study. The first group were chronically lonely from childhood onwards, with reports of loneliness at every stage of life, although a few indicated they were doing better at this stage of older adulthood. The themes among this group included dysfunctional or interrupted parental bonding or attachment, whether through loss, separation of a poor relationship with a present parent, difficulties with close friendships throughout life, alcohol as a coping mechanism, and a consistent inability to be an authentic self and connect with others, although this somewhat resolved for some in older adulthood. While the themes of interrupted of dysfunctional parental bond/attachment, difficulties with close friendships, and inability to be an authentic self or connect to others applied to all in this group, alcohol as a coping mechanism was less universal, as was improved coping in later life. For the second group, they became chronically lonely after they failed to fully recover from some specific change in midlife and still feel lonely now. Among this group, the overarching theme was ‘a life changing event’, which for most was related to clusters of losses or complicated grief, but in one case, this was related to their first episode of mental illness. Also universal among this group was reliance on a small network, which was usually a personal preference, although it has made some losses particularly difficult for these participants. For the third group, loneliness remained situational/transitional only and never became chronic. This group frequently experienced natural periods of loneliness around bereavements and losses; however, they were able to recover from these losses and adapt or cope, much like they also managed in response to other contextual loneliness cues, including sibling gaps, being away from family, lacking financial resources, the COVID-19 pandemic, and deteriorations in the health of self and others in life.

### 4.1. Typologies Identified in Relation to Theories of Loneliness

These three typologies fit with Young’s [38] conceptualisation of loneliness over time [38,39]. He described transient or everyday loneliness as transient or occasional lonely moods, which are of little concern to researchers or clinicians. Situational or transitional loneliness, meanwhile, was described as the experience of people who had satisfying relationships until some specific change occurred, such as a loss, separation, or move, which appeared to fit the experiences of both the second and third groups in the study. This situational loneliness, unlike everyday loneliness, can be severely distressing and, where it persists, can become chronic [39], much like it seemed to become the case for group two in the current study. Finally, chronic loneliness is defined as a person lacking satisfactory social relations for two or more years [39], a description which applied to group one in the current study across the life course and to group two following the life changing transition they experienced in midlife. Young proposed that people who are chronically lonely have beliefs and appraisals which are fundamentally unhelpful to them in the quest for meaningful social connection and lead to a perceived inability to have fruitful and satisfying interpersonal relationships, with fears of being rejected or unworthy [38]. This again fits with group one. In relation to group two, it may be that the life shifting change they experience, in the form of multiple losses or a mental illness, actually impacts how they see themselves and others compared to previously. This is an important area for further research attempting to build a further knowledge base for theory on life course loneliness.

### 4.2. Themes Identified in Relation to the Literature on Loneliness

The linking of loneliness to bereavement and losses across typologies in the current study is supported by many previous qualitative studies on loneliness in older adulthood. Studies from Canada, Sweden, Iran, and the US have all noted themes such as living with loss or the fracture, disruption, or isolation from important, intimate, or meaningful relationships as key in the experience of loneliness among older adults [9,12,40,41,42]. The findings of the current study suggested that while bereavement appears to be universally associated with some loneliness, the degree and chronicity of loneliness following bereavement will be related to additional factors such as attachment style and the presence or absence of additional supportive relationships.

While bereavement or loss is understudied in relation to loneliness in younger people, quantitative studies have noted associations between loneliness and parental bonding in adolescents, university students, and young adults up to the age of 31 [43,44,45] and even among older adults aged 50 and over [46], which supports the identification of this theme in the current study in relation to those who were chronically lonely throughout life (group one). We noted, however, that the absence of a parent alone was not universally predictive of chronic loneliness. Rather, it was an amalgamation of difficulties—issues in early attachment or bonding followed by difficulties with friendships and all of this underlined by an ongoing inability to be one’s authentic self and connect with others. The lack of previous research on life course loneliness, both qualitative and quantitative, as well as the tendency for research at discrete life stages to focus on different issues, makes it challenging to contextualise these findings. Some studies also contradict the importance of childhood predictors, with Matthews et al. [47], for instance, finding no association between young adult loneliness and earlier maternal warmth, maternal depression, parental antisocial behavior, and domestic violence in the home. Looking to the literature for predictors of loneliness which appear to be common across the lifespan and might be determinants of more chronic loneliness suggests beliefs and trust may be important. Loneliness is associated with low trust in both children [48] and adults [49]. This tendency towards a lack of trust could for some stem from dysfunctional parental bonding and insecure attachments formed in infancy, as identified in group one, and the existing quantitative literature illustrating the association of the same with loneliness [43,44,45,50,51,52,53,54,55,56], with a lack of trust perhaps leading to or underlying difficulty for these interviewees in being their authentic selves, truly connecting with others, and making and maintaining close friendships.

The connection between loneliness and personality [4,55] supports the idea of certain individuals having a relative tendency toward chronic loneliness throughout their lives. While supporting this idea of the importance of personal characteristics and how this relates to loneliness throughout life, McHugh et al., as in the current study, also noted that in some cases loneliness does seem to diminish somewhat with age [10]. Greater acceptance later in life also came up as a theme in Graneheim and Lundman’s interview study with adults 85 and over [41]. It may be that greater acceptance of oneself and lifeworld later in life helps to attenuate loneliness. Alcohol was used as a coping mechanism for some individuals that were chronically lonely throughout life (group one) and use of alcohol in the context of loneliness has been previously identified in the literature [56,57]. However, similarly, several who had relied on alcohol were now sober, again signifying a better adjustment and more effective coping strategies in later life [13,41].

Group two did not experience chronic loneliness until midlife onwards and this move into chronic loneliness was again the result of a combination of factors. In this group, the key risk factors were a life-changing event or events in midlife, coupled with a lifelong tendency to rely on a smaller social network by choice. This life-changing event was frequently clusters of multiple losses at once or complicated grief, which is unsurprising given the connection between loneliness and complicated grief. Loneliness which does not improve over time after loss has been described as a prognostic indicator of complicated grief reactions amongst widows in particular, as opposed to widowers [58]. All three women in group two experienced losses. For the male interviewee, his move to chronic loneliness related to his first episode of mental illness (Table 1), which fits with the theme of social withdrawal due to mental health previously found by Achterbergh et al. in their meta-synthesis of the experience of loneliness among young adults [21]. Isolation is also a known risk factor for loneliness [1] and the current study suggests that those with smaller networks, even when by preference (as in the case of group two), are at increased risk of loneliness when they experience major life shifts or losses. This also fits with prior research which has reported that reliance on a small network, along with family-focused networks, can increase the risk of loneliness [14].

Several contextual loneliness factors were identified by the participants in group three who experienced only situational/transitional loneliness but never chronic loneliness, including COVID-19 [57]; financial issues [57,59]; caring responsibilities [60]; and the interviewee’s own health [1]. Also noted were the health of others in their life; lack of siblings and separation from siblings; and the experience of prejudice/bullying in one case. Importantly, however, these interviewees in group three were largely able to deal with and adapt to these contextual factors rather than transition into chronic loneliness as a result.

To summarise, group one experienced interrupted or dysfunctional parental bonds and difficulties with close friendships from a young age, which seemed to set in place the foundation for a lifetime of experiencing chronic loneliness and struggling to authentically connect with others. Group two, as the result of a life-changing event in midlife, such as a cluster of losses, coupled with their preference for a small network, projected into chronic loneliness later. Group three, meanwhile, did not tend to describe the interrupted or dysfunctional parental bonds (seen in group one) or the same level of detrimental life-changing transition in midlife coupled with a preference for a small social network (as in group two). Rather, they experienced typical situational and or transitional loneliness with experiences such as bereavement or poor health, but their resilience, outlook, and alternative sources of social support meant they were able to recover from these periods of loneliness, unlike group one and two.

### 4.3. Implications

The findings of the current study suggest the need for early intervention in order to prevent the seeding of chronic loneliness in childhood through early loss and disrupted attachments. Parental bonding is crucial in early life as a protective factor against chronic loneliness throughout the life course [46] and the early loss of a parent or a difficult relationship with a present parent is a key indicator for intervention with children. Where chronic loneliness does develop and is identified later in life, it is likely that the most effective interventions will address maladaptive social cognitions [38,61], i.e., thoughts and beliefs about the self and others, rather than interventions which address more external circumstantial issues.

In relation to moving from situational loneliness to chronic loneliness, this too must be a key target group for interventions [39]. Further research is needed in relation to loneliness interventions, which may be successful for this group, who opt to rely on a smaller network and experience a major shift in midlife, based on the current study. For some, perhaps taking the time to fully process an important loss or change through counselling may be helpful, while for others it may be that new relationships are also needed and support to form new bonds at this stage may also form part of the solution.

Encouragingly, it also appears that for some individuals, loneliness will remain merely contextual and transitional throughout life, with the individual able to adjust and recover from natural periods of loneliness, such as bereavement or other transitory life stages, such as young adulthood, unaided. Finally, importantly, we note that the three groups identified in the current study are not intended to understate the complexity of loneliness but rather provide a lens to understand the narratives and themes which arose in relation to life course loneliness, as well as an avenue for further research and theory development. Future research should seek to examine these typologies and their associations using large-scale secondary datasets, as richer datasets including reporting of loneliness across the life course become available. The meaning and conceptualisation of loneliness for the participants in the current study will also be addressed in a forthcoming paper. In addition, there are also plans in the future to integrate quantitative and qualitative data on loneliness and social isolation in a mixed-methods analysis.

### 4.4. Strengths and Limitations

As this was a qualitative study, findings are not generalizable beyond the study population and conclusions drawn refer to the sample itself, although useful insights have been gained for potential future research directions in relation to life course loneliness in older adult mental health service users. The study was also strengthened by the participation of a high number of service users, with a spread of age, gender, and education level achieved. The use of a pre-interview questionnaire to identify a minimum level of loneliness also assisted in providing a targeted sample for interviews, although we note not all those objectively assessed as lonely reported current loneliness during their interview. The use of telephone interviews for conducting life qualitative interviews, while not our original plan, had its advantages as telephone interviews tend to take less time than face-to-face interviews and can encourage openness because of anonymity. However, this also means missing visual or facial cues and can feel less personal for some participants. Finally, the narrative interview style and exploratory inductive approach to analysis taken gave voice to participants. Allowing them to define and describe their experiences of loneliness across the life course, what it meant to them, and how it affected them throughout their lives. This allowed our interviewees some opportunity to set the agenda [62] concerning what was important to them in relation to their loneliness experiences throughout life, with our analysis pointing to three distinct typologies apparent from the themes identified.

## 5. Conclusions

Loneliness is a very personal experience, and while everyone’s loneliness may be different, what this study illustrates is the commonality of loneliness, especially among mental health service users. Across the three typologies of loneliness, there was a commonality around the significance of bereavement and loss, which has clear implications for setting in place tailored support around such times, although where other triggers are lacking, loneliness around bereavement typically remains transitional only. The life course approach of this research allowed us to identify childhood dysfunctional or interrupted parental bonding, difficulties with making close friendships, and difficulties connecting with others, which had lifelong implications for group one. Those who moved into chronic loneliness all experienced a major life shift in midlife and preferred to rely on a small social network. For the third group for whom loneliness remained situational, prompts were diverse and covered issues established in the literature to be associated with loneliness such as bereavement, poor health, being a carer, and finances. The insights from this research are important to inform both general loneliness services and policy but also specialist mental health services and training. The importance of our social health needs to be taken seriously by society and we need to identify interventions which prevent situational loneliness becoming chronic for those with smaller networks, as well as to prevent the seeding of chronic life course loneliness in childhood. This paper helps to demonstrate that a life course approach to addressing loneliness must be a priority for public health.

## Figures and Tables

**Table 1:** Overview of results by group, theme, and illustrative quotation.

**Group**	**Theme**	**Illustrative Quotation**
Group 1: Chronically lonely from childhood	Interrupted and dysfunctional parental bond/attachment	“I was taken from my home to the hospital and I spent the guts of five or six years in there… With one visit from mother and maybe one of my sisters, once a week for one hour. And nobody, I never got a hug…” [Alex]
		“Mam, left you with no confidence. All she thought was her bottles of stout (alcohol) and her Woodbines (cigarettes), you know? As far as love was concerned, I don’t know”. [Collette]
		“The fact that I could see other people able to socialise in what I would call a normal way and playful way, that seemed to be beyond me and I didn’t have even the opportunity to test that out because I wasn’t let out”. [John]
	Close friendships difficult or lacking	“I think again, I had no friends, no girlfriends, little friends [in childhood]… I kind of felt [as an adult] that I didn’t have a friend—well, a close friend. I mean, I had loads of friends, and as you know now, from what you have heard, I’m a great chatterbox—in a group, I would contribute lengthy. But I never had that real closeness” [Patricia]
	Alcohol as a coping mechanism	“I actually drank here for a few weeks and it was kind of desperation. I used it as a weapon rather than something to enjoy”. [Alex]
	Inability to be authentic self or connect with others	“I would only just tag along with people and people would think I was part of it but I was there but that was all like I mean” [John]
	Later life greater acceptance	“When you’re lonely as an adult, you’ve nobody to run to, no escape, if you know what I mean, from it… but in the last couple of years I got a bit of help, [nurse] and [doctor] have been brilliant”. [Alex]
Group 2: Loneliness became chronic after a life-changing event at midlife	A life changing event	“I remember feeling very lonely when I had hypomania at one stage about 23 years ago and after that depression set in and felt very lonely because I couldn’t make contact with people. I just I was in my own world as such. The isolation caused me to feel loneliness”. [David]
	Clusters of losses/Complicated grief	“I just seem to be surrounded by people who die…Yeah, I’ve had an awful lot of loss… as I got older I started learning about loneliness and loss—what loss and loneliness is… and I think of the people that I have cared for, loved, whatever, and been close to—they’re all on the other side, if you want to put it that way…” [Paula]
	Reliance on a small network	“I’m not a person for loads of friends… I would, I’d say I’m quiet. I don’t mix with too many people. I’m a kind of one or two type, deal with one or two people at a time. I’m not a real crowd kind of person…. I’d no problem whatsoever making friends with people, talking to people or dealing with people” [David]
Group 3: Loneliness remained situational or transitional only and never moved to chronic	Periods of transitional loneliness around bereavements and losses	“I think it was when one of my parents died…I just felt like there was an empty spot there” [Jane]
	Being on your own/Large sibling gap	“I suppose as a young adult. I was the last of five and they were all much older than I was. So, I didn’t really have anyone to talk to. They were all gone and married… I kind of felt cut off from them like, you know. I felt I missed out being part of the gang”. [Jane]
	Separation from siblings	“At times I did feel a bit lonely...well mostly it was—the rest of the family would come down in the summer to my aunt’s place and when they would go back…I would feel a bit lonely” [Tina when sent away to school as a young child]
	Being briefly left behind by friends before they themselves married	“Next thing you look around and two of them got married and are gone... That’s the way it was, you just accepted it...Most fellas got married and moved away… You know, off they went, that was it. So, you didn’t actually go out and replace them” [Peter]
	Fleeting/contextual loneliness/Pandemic	“Ah yeah, I think since the pandemic I have experienced loneliness, this whole thing of being on my own and not having anyone in the house to talk to” [Rebecca]
	Bullying	“Well I did [experience childhood loneliness], because where I was born and reared, were very few Protestants, it was all Catholic people, and when they’d be having games they’d nearly push me aside, and then they’d call me [names and rhymes]” [Dolores]
	Mobility	“I really love being with people but with the wheelchair, you can’t go anywhere, like if they were going anywhere, out on the town for a meal and all that like I couldn’t expect them to you know put up with me like wheeling me around“ [Angela]

## Data Availability

Due to the sensitive nature of this research, the data collected in this study are not available beyond the research team.

## Supplementary Materials

The following supporting information can be downloaded at: https://www.mdpi.com/article/10.3390/healthcare12111133/s1, File S1: Interview guide; File S2: COREQ checklist.
