# Supplementary material for: Attributions of Loneliness—Life Story Interviews with Older Mental Health Service Users
Source: Healthcare (Basel). 2024 May 31;12(11):1133. doi: 10.3390/healthcare12111133 (PMC11171849; doi:10.3390/healthcare12111133)
Supplement: Supplementary file 1 [file healthcare-12-01133-s001.zip › Supplementary File S1 Life Maps Interview Topic Guide.docx]

**The LIfeMAPS Study - Interview Topic Guide**

(1) Ask about their current situation – where they live, who they live with, social networks, religious attendance/beliefs if any; current health and disabilities.

*“Tell me a bit about yourself…you family and friends…and how you like to/generally spend your time”*

(2) Explore their conceptualisation of loneliness

*“What does loneliness mean to you?*

(3) Explore their loneliness and what prevents them connecting with others;

*“Can you tell me a bit about your life and your experiences of loneliness? If you want start by recalling the first time you felt lonely. There is no rush I am interested to hear about everything that’s important to you to share*

First time recall being lonely….other times (Use A-D as prompts)]

*(A) Explore early childhood and family circumstances*

*(B) Adolescence*

*(C) Middle-age*

*(D)Older age.*

*A-D: Prompt on various life-stages if needed. Throughout taking about these life-stages, asking about how they would describe their experiences of loneliness and also, to consider their response to life-events*

(4) Coping

“What happens when you feel Loneliness? What do you do?”

Use E, F, G as prompts:

(E) How do you cope with Loneliness?]

(F) Are there tools you have found?]

(G) Has this changed over time e.g. earlier in your life compared to now?]

(5) Overall what they think would help attenuate their and other people’s loneliness (now and during various age-stages).

“*What do you think would help people who feel lonely?”*

[…your and/or loneliness of other people? Loneliness at earlier time for you]

*Thank for participation and Debrief*

*Please cite as: Burns, A.; Leavey, G.; Lawlor, B.; Golden, J.; Reilly, D.; O’Sullivan, R. Attributions of Loneliness—Life Story Interviews with Older Mental Health Service Users. Healthcare, 2024*.
